# Missed Opportunities for Tetanus Postexposure Prophylaxis — California, January 2008–March 2014

**Published:** 2015-03-13

**Authors:** Cynthia Yen, Erin Murray, Jennifer Zipprich, Kathleen Winter, Kathleen Harriman

**Affiliations:** 1 California Department of Public Health

Tetanus is an acute and sometimes fatal disease characterized by sudden muscle contractions. The number of tetanus cases reported annually in the United States has declined significantly since the 1930s and 1940s as a result of the introduction of tetanus vaccines (1). However, sporadic cases continue to occur in persons who are not up-to-date with tetanus toxoid-containing vaccinations (TT) and do not receive appropriate postexposure prophylaxis (PEP). To assess the extent of these cases, the California Department of Public Health reviewed all tetanus cases reported during January 2008–March 2014. A total of 21 tetanus patients were reported; five (24%) died. An average of three cases were reported each year during 2008–2013; the average annual incidence among patients aged ≥65 years (0.23 cases per 1 million population) was twice that among patients aged 21–64 years (0.10 cases per 1 million population). Of 16 patients with an acute injury before illness and diagnosis, nine (56%) sought medical care, and two (22%) of the nine received appropriate PEP. Although tetanus is rare, it is a life-threatening disease that is preventable. Health care providers should ensure that their patients are up-to-date with TT vaccination and provide appropriate postexposure prophylaxis for patients with wounds.

During 2008–2010, a confirmed case was defined by the Council of State and Territorial Epidemiologists (CSTE) as a patient with acute onset of hypertonia or painful muscular contractions (usually of the muscles of the jaw and neck) and generalized muscle spasms without other apparent medical cause.* In 2010, CSTE removed the “confirmed” classification and defined all clinically compatible cases as probable.† The California Department of Public Health analyzed all confirmed and probable cases in accordance with CSTE case definitions. Using the CDC tetanus surveillance worksheet, local health department and California Department of Public Health staff reviewed case surveillance and medical record data, including demographics, clinical presentation and course, vaccination status, and wound management. Vaccination and wound data were reviewed to determine whether health care providers followed wound management and PEP recommendations (2,3). Tetanus incidence rates were calculated using population estimates from the California Department of Finance. Hospitalization costs were estimated using discharge data from the Office of Statewide Health Planning and Development.

During January 2008–March 2014, a total of 21 tetanus cases were reported; five (24%) were fatal (Table 1). The patients were all adults ranging in age from 21 to 89 years (median = 52 years); 15 (71%) were male. An average of three cases were reported each year during 2008–2013 (range = 0–5). The average annual tetanus incidence rate during 2008–2013 was 0.09 cases per 1 million population, compared with 0.19 cases during 2002–2007. During 2008–2013, the average annual incidence among patients aged ≥65 years (0.23 cases per 1 million population) was twice that among patients aged 21–64 years (0.10 cases per 1 million population). The case-fatality rate among patients aged ≥65 years was 50%, compared with 13% among patients aged 21–64 years. Race and ethnicity were reported for 18 (86%) patients. The average annual incidence rates among Hispanics (0.08 cases per 1 million population), non-Hispanic whites (0.09), non-Hispanic blacks (0.07), and non-Hispanic Asians/Pacific Islanders (0.03) were similar.

All 21 tetanus patients were hospitalized; 19 (90%) were admitted to an intensive care unit, and nine required mechanical ventilation. The median number of days hospitalized was 18 (range = 2–65); of 15 patients for whom data were available, the median cost of total hospital charges per patient was $166,259 (range = $22,229–$1,024,672). Seven patients had conditions associated with increased risk for tetanus; four were diabetic, and three were injection-drug users (1).TT history was reported for 12 (57%) patients; three (25%) could not recall receiving any doses, and nine (75%) recalled receiving ≥1 dose. Among the nine patients who recalled receiving ≥1 dose, six received their last dose 10 to 50 years before their illness, and three could not recall when they received their last dose.

Sixteen (76%) patients reported that an acute injury had occurred before illness onset; including punctures (seven), abrasions (four), linear lacerations (three), compound fracture (one) and animal bite (one). Of six patients with data on wound depth, two had wounds that were >1 cm deep. Seven of 11 patients with available data had wounds that appeared infected, and two of seven patients with available data had wounds with devitalized, ischemic, or denervated tissue. Five patients reported no acute injuries before onset; of these, three were injection-drug users. The remaining two patients could not recall any acute injuries; however, one reported an insect bite, and the other reported chronic abrasions on the hands and feet and exposure to soil.

Of the 16 patients who reported acute injuries before illness onset, nine had sought medical care for their injuries (Table 2). Of the nine, only two received appropriate PEP before the onset of tetanus symptoms as recommended by the Advisory Committee on Immunization Practices (ACIP) (Table 3) (2,3). Of the seven patients who did not receive appropriate PEP, five had punctures or contaminated wounds and unknown TT vaccination histories, and should have received both TT and tetanus immune globulin (TIG) as recommended. However, four patients did not receive any PEP, and one received TT PEP only. Of the two remaining patients, one had a clean, minor wound and reported receiving at least one TT dose more than 10 years ago, but was not offered TT PEP as recommended; the other patient was contraindicated for TT because of a history of anaphylaxis, but was not offered TIG as an alternative.

Following their tetanus diagnoses, all 21 patients were treated with TIG; six were treated ≤1 day after symptom onset, eight ≤4 days, six ≤9 days, and one was treated >2 weeks after onset. Among the five fatal cases, one patient was treated ≤1 day after symptom onset, two were treated ≤4 days, and two ≤9 days after onset. Of 15 patients for whom data on TIG dosage were available, five received less than the 3,000–6,000 U that is generally recommended for treatment (4); two received less than 500 U, and three received 500–1,000 U.

## Discussion

Although rates of tetanus have declined, sporadic cases continue to occur, particularly in adults who are not up-to-date with TT. Vaccination coverage among children is higher; in 2012, an estimated 82.5% of U.S. children aged 19–35 months and 84.6% of U.S. children aged 13–17 years had received ≥4 doses of diphtheria toxoid-tetanus toxoid-acellular pertussis vaccine (DTaP) and ≥1 dose of tetanus, diphtheria, acellular pertussis vaccine (Tdap), respectively (5,6). In contrast, only 62% of adults aged ≥19 years had received TT during the preceding 10 years; coverage for adults aged ≥65 years was 55% (7). All of the tetanus patients reported in California during January 2008–March 2014 were adults aged ≥21 years. Among the 12 patients with verified vaccination histories, none recalled receiving TT during the preceding 10 years. Health care providers should assess patient vaccination status during routine visits to determine whether TT is needed. ACIP recommends that after receiving a primary childhood series, a tetanus and diphtheria vaccine (Td) dose should be given during adolescence and every 10 years thereafter. For added protection against pertussis, one of the Td booster doses should be Tdap if it was not previously administered (2,3).

Even minor wounds or abrasions can result in tetanus, highlighting the importance of ensuring that patients are up to date for TT (8,9). Providers should assess vaccination status in patients with wounds and in particular older adults, injection-drug users, patients with diabetes, and those with chronic wounds, all of whom are considered at increased risk for tetanus (1). Patients who have completed the 3-dose primary TT series need a booster dose as part of wound management if they have a clean, minor wound and received their last TT dose more than 10 years prior to injury, or if they have any other type of wound and received their last TT dose more than 5 years prior to injury (2). ACIP recommends that persons with unknown or incomplete histories receive TT as part of routine wound management; patients with wounds that are neither clean nor minor should receive TIG in addition to TT. Although the dosage of TIG for PEP is not specified in the recommendations, dosage information is provided in the product insert.§

In this analysis, only nine of 16 patients with acute injuries had sought medical care before their tetanus illness onset and diagnosis, and only two of the nine received PEP with TT or TT plus TIG as recommended (2–4). Health care providers might fail to provide TIG PEP because of a lack of knowledge about current recommendations or because the assessment of wound severity and whether a patient should be managed with TIG according to ACIP recommendations can be subjective (2,10). All tetanus patients required considerable and costly medical care, including hospitalization, and almost all (90%) were admitted to intensive care. Among patients who received TIG as treatment, there was variability in the dose administered. In the United States, 3,000–6,000 U, given in a single intramuscular dose with part of the dose infiltrated around the wound if it can be identified, is generally recommended for treatment. However, the optimal therapeutic dose has not been established, and some experts contend that a dose of 500 U, as recommended by the World Health Organization,¶ is as effective as higher doses and causes less discomfort (4). It is also possible that some providers treating tetanus patients inadvertently prescribed the PEP dosage of TIG rather than the treatment dosage. Among five treated patients who received <3,000 U of TIG as treatment, three survived and two died. Among 10 treated patients who received ≥3,000 U of TIG, seven survived, and three died.

The findings in this report are subject to at least three limitations. First, although California health care providers are required to report tetanus cases, surveillance is passive, and underreporting is likely. Second, because there is no laboratory testing for tetanus and case identification depends solely on clinical assessment, some cases might be misclassified. Finally, some of the case reports were incomplete, particularly with regard to TT history.

Although significant progress has been made in reducing the morbidity and mortality caused by tetanus, cases of this vaccine-preventable disease continue to be reported. Health care providers should assess the tetanus vaccination status of their patients during routine visits. All providers who provide care for patients with wounds should have protocols for tetanus PEP and ensure that appropriate PEP is provided for such patients.

What is already known on this topic?

The incidence of tetanus has declined significantly since the introduction of tetanus vaccines. However, sporadic cases continue to be reported, particularly in adults who are not up-to-date with tetanus vaccinations.

What is added by this report?

During January 2008–March 2014 in California, a total of 21 tetanus patients were reported. All were hospitalized, including 19 in intensive care units; five (24%) died. Of 16 patients with an acute injury prior to illness, only nine had sought medical care, and only two of the nine received appropriate postexposure prophylaxis. In addition, some patients with tetanus were not administered the recommended dosage of tetanus immune globulin.

What are the implications for public health practice?

Routine vaccination of patients every 10 years is important to prevent tetanus, particularly in settings where patients do not seek medical care following an injury, where no injury is evident to the patient, or where appropriate postexposure prophylaxis is not provided following an injury. Efforts to educate health care providers might lead to better tetanus postexposure prophylaxis for patients with wounds and better use of therapeutic tetanus immune globulin for patients with tetanus.

## Figures and Tables

**TABLE 1:** Number of tetanus cases (N = 21), by selected characteristics and outcome — California, January 2008–March 2014

	Died		Survived		Total	
			
Characteristic	No.	(%)	No.	(%)	No.	(%)
**Sex**						
Male	3	(60)	12	(75)	15	(71)
Female	2	(40)	4	(25)	6	(29)
**Age group (yrs)**						
21–49	2	(40)	8	(50)	10	(48)
50–64	0	—	5	(31)	5	(24)
≥65	3	(60)	3	(19)	6	(29)
**Clinical course**						
Hospitalized	5	(100)	16	(100)	21	(100)
Admitted to ICU	5	(100)	14	(88)	19	(90)
Median no. of days hospitalized (range)	19 (4–38)		17 (2–65)		17 (2–65)	
**Underlying conditions**						
Diabetes	1	(33)	3	(19)	4	(19)
Injection drug user	0	—	3	(19)	3	(14)
**Tetanus vaccination history**						
Zero doses	2	(40)	1	(6)	3	(14)
At least one dose*	1	(20)	8	(50)	9	(43)
Unknown	2	(40)	7	(44)	9	(43)
**Injury history**						
Acute injury before illness	5	(100)	11	(69)	16	(76)
Puncture	2	(40)	5	(45)	7	(44)
Abrasion	1	(20)	3	(27)	4	(25)
Linear laceration	1	(20)	2	(18)	3	(19)
Compound fracture	1	(20)	0	—	1	(6)
Animal bite	0	—	1	(9)	1	(6)
Sought medical care for acute injury	5	(100)	4	(36)	9	(56)
Received recommended postexposure prophylaxis	1	(20)	1	(25)	2	(22)

* Of patients who had received at least one dose of TT-containing vaccine, none recalled receiving a dose in the preceding 10 years.

**TABLE 2:** Therapeutic treatment of tetanus patients (n = 9) with an acute wound who had sought medical care before illness and diagnosis — California, January 2008–March 2014

Patient No.	Age	Sex	TT history	When last dose received	Wound type	Received TT	Received appropriate PEP	Units of therapeutic TIG received	Outcome
1	48	Female	Unknown	Unknown	Abrasion	Yes	Yes	5,000	Survived
2	38	Male	Zero doses	N/A	Linear laceration	Yes	Yes	5,000	Died
3	73	Male	At least one dose	Unknown	Compound fracture	No*	No	1,000	Died
4	45	Male	Unknown	Unknown	Puncture	No	No	250	Died
5	47	Female	Unknown	Unknown	Animal bite	No	No	500	Survived
6	68	Male	Unknown	Unknown	Puncture	No	No	Unknown	Survived
7	71	Male	At least one dose	50 years ago	Abrasion	No	No	Unknown	Survived
8	86	Female	Zero doses	N/A	Puncture	No	No	3,000	Died
9	89	Female	Unknown	Unknown	Abrasion (contaminated)	Yes	No	5,000	Died

**Abbreviations:** TT = tetanus toxoid; TIG = tetanus immune globulin; N/A = not applicable.

* Patient reported allergy to TT.

**TABLE 3:** Recommended management of tetanus wounds — Advisory Committee on Immunization Practices

	Clean, minor wounds		All other wounds	
		
Vaccination history	Administer Td*	Administer TIG	Administer Td*	Administer TIG
Unknown or <3 doses	Yes	No	Yes	Yes
≥3 doses	No†	No	No§	No

**Abbreviations:** Td = tetanus and diphtheria vaccine; TIG = tetanus immune globulin.

**Sources:** CDC. Preventing tetanus, diphtheria, and pertussis among adults: use of tetanus toxoid, reduced diphtheria toxoid and acellular pertussis vaccine (Tdap). Recommendations of the Advisory Committee on Immunization Practices (ACIP) and recommendations of ACIP, supported by the Healthcare Infection Control Practices Advisory Committee (HICPAC), for use of Tdap among health-care personnel. MMWR Recomm Rep 2006;55(No. RR-17).

CDC. Updated recommendations for use of tetanus toxoid, reduced diphtheria toxoid, and acellular pertussis (Tdap) vaccine in adults aged 65 years and older—Advisory Committee on Immunization Practices (ACIP), 2012. MMWR Morb Mortal Wkly Rep 2012;61:468–70.

* Tdap (tetanus, diphtheria, acellular pertussis vaccine) may be substituted for Td if the person has not previously received Tdap and is aged ≥10 years.

† Yes, if >10 years since last dose.

§ Yes, if >5 years since last dose.

## Footnotes

* Additional information available at http://wwwn.cdc.gov/NNDSS/script/casedef.aspx?CondYrID=864&DatePub=1/1/1996.

† Additional information available at http://wwwn.cdc.gov/NNDSS/script/casedef.aspx?CondYrID=865&DatePub=1/1/2010.

§ Additional information available at http://www.talecris-pi.info/inserts/BayTet.pdf.

¶ Additional information available at http://www.who.int/diseasecontrol_emergencies/who_hse_gar_dce_2010_en.pdf.

## References

[1] CDC (2011). Tetanus surveillance—United States, 2001–2008. MMWR Morb Mortal Wkly Rep.

[2] CDC (2006). Preventing tetanus, diphtheria, and pertussis among adults: use of tetanus toxoid, reduced diphtheria toxoid and acellular pertussis vaccine (Tdap). Recommendations of the Advisory Committee on Immunization Practices (ACIP) and recommendations of ACIP, supported by the Healthcare Infection Control Practices Advisory Committee (HICPAC), for use of Tdap among health-care personnel. MMWR Recomm Rep.

[3] CDC (2012). Updated recommendations for use of tetanus toxoid, reduced diphtheria toxoid, and acellular pertussis (Tdap) vaccine in adults aged 65 years and older—Advisory Committee on Immunization Practices (ACIP), 2012. MMWR Morb Mortal Wkly Rep.

[4] Pickering LK, Baker CJ, Kimberlin DW, Long SS, American Academy of Pediatrics (2012). Tetanus. Red Book: 2012 Report of the Committee on Infectious Diseases.

[5] CDC (2013). National, state, and local area vaccination coverage among children aged 19–35 months—United States, 2012. MMWR Morb Mortal Wkly Rep.

[6] CDC (2013). National and state vaccination coverage among adolescents aged 13–17 years—United States, 2012. MMWR Morb Mortal Wkly Rep.

[7] Williams WW, Lu P-J, O’Halloran A (2014). Noninfluenza vaccination coverage among adults—United States, 2012. MMWR Morb Mortal Wkly Rep.

[8] Pascual FB, McGinley EL, Zanardi LR, Cortese MM, Murphy TV (2003). Tetanus surveillance—United States, 1998–2000. MMWR Surveill Summ.

[9] Rhee P, Nunley MK, Demetriades D (2005). Tetanus and trauma: a review and recommendations. J Trauma.

[10] McVicar J (2013). Should we test for tetanus immunity in all emergency department patients with wounds?. Emerg Med J.

